# Sympathoactivation and rho-kinase-dependent baroreflex function in experimental renovascular hypertension with reduced kidney mass

**DOI:** 10.1186/1472-6793-14-4

**Published:** 2014-06-19

**Authors:** Rainer U Pliquett, Sebastian Benkhoff, Oliver Jung, Ralf P Brandes

**Affiliations:** 1 Institute for Cardiovascular Physiology, Vascular Research Centre, Fachbereich Medizin, Goethe University, Frankfurt (Main), Germany; 2 Department of Nephrology, Clinic of Internal Medicine 2, University Clinic Halle, Martin Luther University Halle-Wittenberg, Ernst-Grube-Str. 40, Halle (Saale) 06120, Germany; 3 Department of Nephrology, Goethe University, Frankfurt (Main), Germany

**Keywords:** Arterial hypertension, Sympathetic nervous system, Baroreflex, Irbesartan

## Abstract

**Background:**

Dysregulation of the autonomic nervous system is frequent in subjects with cardiovascular disease. The contribution of different forms of renovascular hypertension and the mechanisms contributing to autonomic dysfunction in hypertension are incompletely understood. Here, murine models of renovascular hypertension with preserved (2-kidneys-1 clip, 2K1C) and reduced (1-kidney-1 clip, 1K1C) kidney mass were studied with regard to autonomic nervous system regulation (sympathetic tone: power-spectral analysis of systolic blood pressure; parasympathetic tone: power-spectral analysis of heart rate) and baroreflex sensitivity of heart rate by spontaneous, concomitant changes of systolic blood pressure and pulse interval. Involvement of the renin-angiotensin system and the rho-kinase pathway were determined by application of inhibitors.

**Results:**

C57BL6N mice (6 to 11) with reduced kidney mass (1K1C) or with preserved kidney mass (2K1C) developed a similar degree of hypertension. In comparison to control mice, both models presented with a significantly increased sympathetic tone and lower baroreflex sensitivity of heart rate. However, only 2K1C animals had a lower parasympathetic tone, whereas urinary norepinephrine excretion was reduced in the 1K1C model. Rho kinase inhibition given to a subset of 1K1C and 2K1C animals improved baroreflex sensitivity of heart rate selectively in the 1K1C model. Rho kinase inhibition had no additional effects on autonomic nervous system in either model of renovascular hypertension and did not change the blood pressure. Blockade of AT1 receptors (in 2K1C animals) normalized the sympathetic tone, decreased resting heart rate, improved baroreflex sensitivity of heart rate and parasympathetic tone.

**Conclusions:**

Regardless of residual renal mass, blood pressure and sympathetic tone are increased, whereas baroreflex sensitivity is depressed in murine models of renovascular hypertension. Reduced norepinephrine excretion and/or degradation might contribute to sympathoactivation in renovascular hypertension with reduced renal mass (1K1C). Overall, the study helps to direct research to optimize medical therapy of hypertension.

## Background

Nephrogenic arterial hypertension comprising renovascular and renoparenchymal aetiologies is increasingly prevalent [1]. Hypertensive patients with chronic kidney disease (CKD) are three times more likely to die within 8 years than hypertensive counterparts without CKD [2]. Regarding renovascular hypertension, revascularization strategies do not convey any benefit when compared to the best conservative therapy [3,4]. Angiotensin II-subtype-1 (AT1) receptor blockers [5] or angiotensin-converting enzyme (ACE) inhibitors [6] slow the progression of CKD, yet they are contraindicated in bilateral renal artery stenosis or in unilateral renal artery stenosis and (functional) single kidney situation. Given the constraints inherent to medical and interventional therapies of renovascular hypertension, novel therapeutic targets are still needed.

The autonomic nervous system is such a potential target. Baroreflex function is attenuated in renovascular disease, regardless of residual kidney mass [7]. On the basis of the effect of propranolol and atropine methyl nitrate on resting heart rate, an elevated sympathetic tone in models of renovascular hypertension with (1-kidney-one-clip; 1K1C) and without kidney-mass reduction (2-kidneys-one-clip; 2K1C) was identified [8,9]. Aside from heart-rate changes, muscle sympathetic nerve activity [10] and functional data like cold-pressor test [11] were not affected by propranolol. Therefore, additional surrogates of sympathetic tone are needed.

The pathomechanism of sympathoactivation in renovascular hypertension is unclear. In experimental renovascular hypertension with preserved kidney mass (2K1C), the renin-angiotensin-aldosterone system (RAAS) is found to be activated [12], and central nervous system effects of angiotensin II probably are the driving force of sympathoactivation [13]. In experimental renovascular hypertension with reduced kidney mass (1K1C), however, the RAAS is suppressed [12], and other sympathoactivating pathomechanisms must be operative.

The intracellular Rho A/Rho kinase system emerges as a novel target for the treatment of cardiovascular disease [14]. Rho A, a small GTPase, has numerous functions and is involved in cytoskeletal organization. Upon activation, Rho A interacts with and activates the Rho A-dependent kinase (ROCK). As a consequence endothelial nitric oxide synthase mRNA is destabilized and cellular contraction is initiated by means of calcium-sensitization [15] which also increases endothelial cell permeability [16].

Established Rho A/ROCK inhibitors are Fasudil but also statins, 3-hydroxy-3-methylglutaryl-Coenzyme A reductase inhibitors. Statins inhibit the formation of geranyl-geranylpyrophosphate, a prerequisite for RhoA membrane anchoring [14,17]. We have previously shown that simvastatin lowers sympathetic tone in experimental chronic heart failure, another condition characterized by sympathoexcitation [18,19]. Also ROCK inhibition (ROKI) by Fasudil was shown to improve baroreflex sensitivity in experimental chronic heart failure when given in to the intracerebroventricular space [20]. This effect was blunted by intracerebroventricular application of L-NAME, an inhibitor of endothelial nitric oxide synthase, suggesting direct central effects and a contribution of central nitric oxide in this process. The value of ROCK inhibition for the treatment of hypertension at large, however, is still unclear.

In the present study, we hypothesize that sympathoactivation is more pronounced in renovascular hypertension with preserved (2K1C) versus reduced (1K1C) kidney mass when using heart-rate independent surrogates of sympathetic tone. Sham surgery animals and irbesartan (Irb)-treated 2K1C animals were used as control groups. The AT1-receptor blocker treatment was used as a positive control for its sympathoinhibitory actions [21-24]. Secondly, we hypothesize that ROKI enhances baroreflex sensitivity of heart rate in models of renovascular hypertension (1K1C; 2K1C) in analogy to the chronic-heart failure situation [20].

## Methods

### Animals

Male C57BL/6 N mice (6–11 per group, age: 10–12 weeks, Charles River, Sulzfeld, Germany) were housed in individual cages in a separate room under standard conditions (21°C, 12 h dark–light cycle), standard chow and drinking water ad libitum. Care was provided daily at the same time, body weight was taken weekly. All animal procedures and experiments adhered to the APS’s Guiding Principles in the Care and Use of Vertebrate Animals in Research and Training. During surgeries, inhalational anaesthesia using a precision vaporizer with isoflurane (2% initially, 0.8–1% continuously in an oxygen stream of 0.2 l/min), and subcutaneous (SC) fentanyl (0.06 mg/kg) were used. Following surgeries, pain-relief medication buprenorphine (0.3 mg/kg SC), and antibiotic prophylaxis with ampicillin (50 mg/kg SC) were administered. After observing the animals for 4–6 weeks, mice were sacrificed (isoflurane anaesthesia, decapitation), and heart weight (absolute and relative to body weight) was determined. Ethical approval was obtained from local animal-care officials and the supervising federal authority (approval number: V54-19c20/15F28K2154 issued by Regierungspräsidium Darmstadt, Hesse, Germany).

### Telemetric monitoring

Aortic blood pressure of unrestrained, conscious mice was monitored by telemetry (telemetry unit: TA11PA-C10, Data Sciences International, St. Paul, Minnesota, USA) attached to a femoral-artery catheter. For catheter placement, a 15 mm skin incision was made, and the left femoral artery and vein were separated using a non-serrated fine-tip forceps (Dumont®, Roboz Surgical Instrument Co. Inc., USA). The left femoral artery was tied off (PERMA-HAND® silk, Ethicon, USA) caudally of the superficial epigastric and superficial circumflex iliac artery. A second tie was placed 10 to 12 mm cranially and kept under tension to stop perfusion. A 90°-bent 26 gauge injection needle serving as a catheter introducer was inserted into the left common femoral artery right above the distal tie. The telemetry catheter was inserted and advanced to the proximal tie. After releasing the proximal tie temporarily, the catheter was further advanced into the lower aorta (below the renal artery) and secured by two knots. After freeing a subcutaneous pouch on the right flank, the transmitter unit connected to the intra-arterial catheter was inserted and 0.05 – 0.1 g gentamicin solution (3 mg/g) was applied before skin suture (4–0 Prolene, Ethicon, USA). Blood pressure readings were transmitted to a receiver placed below the mouse cage, digitized with a sampling rate of 1000 Hz and stored and analyzed on a workstation in a separate room. Systolic and diastolic blood pressures, pulse pressure and pulse intervals (defined as consecutive dP/dt) were extracted from aortic blood pressure waveforms using ART 4.2 Gold software (Data Sciences International; St. Paul, Minnesota, USA). One week later, the mice were randomized in a 1:2 fashion to sham surgery (normal controls) or unilateral renal-artery stenosis, i.e. the 2K1C model of hypertension with preserved kidney mass. There, a U-shaped metal clip (Exidel SA, Switzerland; width: 110 ± 0.07 μm) was implanted around the right renal artery as reported previously [12,25]. One week later, every second 2K1C mouse was subjected to nephrectomy of the non-clipped kidney yielding the 1K1C model of renovascular hypertension with reduced kidney mass.

### Autonomic nervous system testing

After a two-week recovery period, a one-hour baseline recording was taken in the morning. Thereafter, intraperitoneal injections of atropine-methyl nitrate (ATR, 2 mg/kg in 4 ml/kg saline, Sigma) [26] or metoprolol (MET, 1 mg/kg in 4 ml/kg saline; Sigma) were performed. ATR was used to block the parasympathetic component while MET was used to block the sympathetic component of the autonomic nervous system. After injection of either substance, another hour of continuous blood-pressure recording was performed. For each one-hour recording, the last 30 minutes were used for analysis. Mean heart rate and blood pressure were determined. In addition, consecutive, continuous one-minute series of digitized systolic blood pressure and pulse-interval data were linearly interpolated with an equidistant sampling interval of 0.05 s (20 Hz). Power spectral analysis of those systolic blood pressure and pulse intervals was performed using Fourier transformation (1024-point series corresponding to a 51.2-s period). Each spectral band obtained was a harmonic of 20/1024 Hz (0.019 Hz). The power spectral analysis of blood pressure and pulse intervals yielded intensities (units: mmHg^2^ and ms^2^) for a given spectral bandwidth. The cumulative intensity of the low-frequency band (0.15-0.6 Hz) of power spectrum of systolic blood pressure (LF-SBP) was regarded as a quantitative measure of sympathetic tone, whereas the cumulative intensity of the high-frequency band (2.5-5.0 Hz) of power spectrum of heart rate (HF-HRV) was considered as a quantitative measure of parasympathetic tone [26,27].

Power spectrum (high-frequency band: 1.0-5 Hz) of systolic blood pressure (HF-SBP) [28,29] and power spectrum (low-frequency band: 0.4-1.5 Hz) of heart rate (LF-HRV) were provided as supplemental data. With regard to sympathovagal balance, interpretation of HF-SBP data still remains inconclusive for the mouse model. However, in contrast to humans, LF-HRV is considered to be an alternative quantitative measure of parasympathetic tone in mice [30].

In addition, changes in resting heart rate after administration of metoprolol or atropine were determined. An overnight recovery was required after injection of either substance.

### Baroreflex sensitivity

Baroreflex sensitivity was determined by the sequence technique [31] of concomitant changes of systolic blood pressure and pulse intervals (digitized, linearly interpolated) utilizing the Hemolab software (http://www.haraldstauss.com/HemoLab/HemoLab.php). Concomitant changes of systolic blood pressure (of at least 15 mmHg) and pulse intervals of at least 4 consecutive heart beats were correlated. For individual baroreflex curves, a correlation coefficient of at least 0.9 was mandated for analysis. In addition, a time delay of 0 seconds was chosen for analysis of concomitant blood-pressure and pulse-interval changes according to a previous study with murine models [32]. The average of at least 10 individual baroreflex slopes (linear portion of systolic blood pressure – pulse-interval relationship; unit: ∆bpm/∆mmHg) was considered as baroreflex sensitivity.

### Urinary catecholamine assay

Mice were placed in metabolic cages (Tecniplast) for 24-hour urine collection. Urine was collected over 24 hours in a vial containing 30 μl HCl (0.5 mol/l), stored at −20°C. For analysis urinary norepinephrine, dopamine and epinephrine were determined by a radioimmunoassay method (LDN 3-CAT RIA, Labor Diagnostika Nord, Nordhorn, Germany).

### Medical intervention

The AT1 receptor blocker irbesartan **(**Irb) was dissolved in water (c = 150 mg/l, projected dose: 30 mg/kg/d) and given to five 2K1C mice orally ad libitum. Drug uptake was recorded by weighing drinking bottles every 48 h. Likewise, the Rho-kinase inhibitor SAR407899A was given to 2K1C and 1K1C mice with the drinking water (c = 50 mg/l, projected dose: 10 mg/kg/d). The actual drug uptake is shown in Table 1. Experiments were carried out after 7 – 9 days on treatment and compared to normal controls and to 2K1C animals without treatment. Irbesartan and the Rho-kinase inhibitor SAR407899A were kindly provided by Sanofi-Aventis, Frankfurt/Main, Germany.

**Table 1:** Drug uptake with the drinking water (mg/kg/d) in renovascular-hypertension models: 2-kidney-1-clip, 1-kidney-1-clip

	**2-kidneys-1-clip model of hypertension**	**1-kidney-1-clip model of hypertension**	**P**
Rho-kinase inhibitor SAR407899A	10.5 ± 2.4	10.9 ± 3.5	ns
Irbesartan	26.8 ± 2.0	NA	NA

Statistical analysis was performed between groups.

### Statistics

Results are given as means ± one standard deviation. For inter-group comparisons with equal variances, one-way ANOVA/Newman-Keul post-hoc test or one- or two-tailed student’s t-test were used, where appropriate. If the normality test failed, nonparametric tests (Kruskal Wallis test/Dunn’s post-hoc test or – for two-groups - Mann–Whitney-U or Wilcoxon-matched pairs test) were used, where appropriate. A p < 0.05 was considered significant. Asterisks highlight significances (*p < 0.05; **p < 0.01; ***p < 0.001). Statistical analysis was carried out with Graphpad (La Jolla, California, USA).

## Results

### Characteristics of models of renovascular arterial hypertension (2K1C, 1K1C)

**Figure 1:**
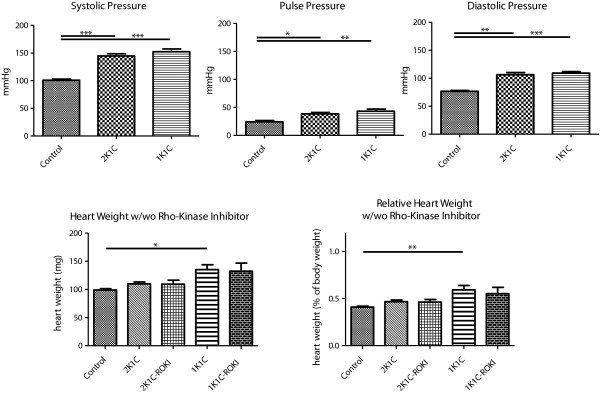
Systolic and diastolic blood pressures and pulse pressure in controls (Control) and in animals with renovascular hypertension (1K1C, 2K1C); absolute and relative heart weights of Control, 1K1C and 2K1C animals with or without prior Rho-kinase inhibitor (ROKI) treatment (SAR407899A; average uptake: 10.6 ± 2.3 mg/kg/d).

Pulse pressure, systolic and diastolic blood pressure were significantly elevated in both models (2K1C, 1K1C) of renovascular hypertension as compared to controls (Figure 1, upper panel). Treatment with the Rho-kinase inhibitor SAR407899A did not affect blood pressure (data not shown). In comparison to controls, 1K1C animals also exhibited an increase in both absolute and relative heart weight. This difference in heart weight was not observed after ROKI (SAR407899A) treatment (Figure 1, lower panel). As the increase in heart weight indicates left-ventricular hypertrophy, this finding suggests that ROKI treatment attenuates 1K1C-associated left-ventricular hypertrophy.

**Figure 2:**
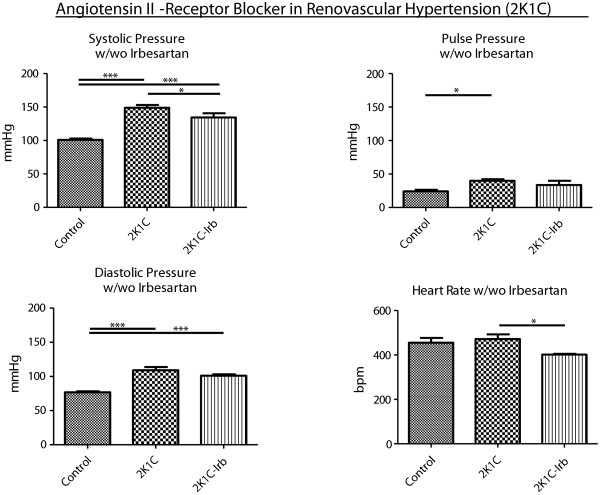
Systolic and diastolic blood pressures, pulse pressure and heart rate in controls (Control) and in renovascular-hypertension animals with preserved kidney mass (2K1C) with and without irbesartan (Irb) treatment (Irb uptake: 26.8 ± 2.0 mg/kg/d).

Heart rate was not different among groups at baseline or following selective autonomic-nervous-system blockade with atropine (ATR) or metoprolol (MET) (Table 2). However, resting heart rate significantly increased in response to ATR in all groups. In the control group, ATR also increased the diastolic blood pressure. Neither pulse pressure nor systolic blood pressure were affected by ATR or MET in any group. To gauge autonomic nervous system effects of an AT1 blockade, Irb treatment was performed in 2K1C animals. Hemodynamic effects of Irb included a decrease of both systolic blood pressure and heart rate in 2K1C animals (Figure 2).

**Table 2:** Baseline characteristics of renovascular-hypertension models (2-kidney-1-clip, 1-kidney-1-clip), and of normal controls following sham-surgery

	**Normal controls**	**2-kidneys-1-clip model of hypertension**	**1-kidney-1-clip model of hypertension**	**P**
Heart rate (bpm)	455.3 ± 60.8	472.5 ± 54.9	463.6 ± 55.4	ns
Systolic blood pressure - baseline (mmHg)	100.9 ± 5.5	144.8 ± 13.0	152.3 ± 16.6	<0.0001
Diastolic blood pressure - baseline (mmHg)	76.7 ± 4.3	106.4 ± 12.7	109.2 ± 8.7	0.0001
Heart rate – after atropine (bpm)	559.8 ± 58.1	575.9 ± 82.5	604.4 ± 55.4	ns
Systolic blood pressure - after atropine (mmHg)	108.9 ± 6.6	152.7 ± 16.1	151.2 ± 15.3	<0.0001
Diastolic blood pressure - after atropine (mmHg)	85.9 ± 6.0	116.0 ± 11.9	111.2 ± 8.4	<0.0001
Heart rate – after metoprolol (bpm)	463.0 ± 74.0	513.8 ± 67.6	479.3 ± 49.4	ns
Systolic blood pressure - after metoprolol (mmHg)	100.2 ± 10.8	155.1 ± 14.9	151.1 ± 15.3	<0.0001
Diastolic blood pressure - after metoprolol (mmHg)	77.3 ± 9.2	114.1 ± 13.4	111.4 ± 10.5	<0.0001

Statistical analysis was performed among groups.

### Baroreflex sensitivity of heart rate is attenuated in models of renovascular hypertension, irrespective of residual renal mass (2K1C, 1K1C)

**Figure 3:**
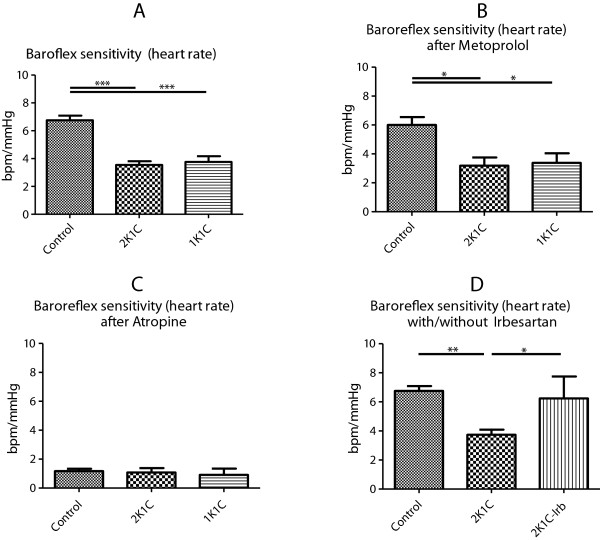
Baroreflex sensitivity of heart rate in controls (Control), 1K1C, and 2K1C animals (A); in Control, 1K1C, and 2K1C animals following MET (B); in Control, 1K1C, and 2K1C animals following ATR (C); in Control and in irbesartan (Irb)-treated and untreated 2K1C animals (D).

In comparison to controls, baroreflex sensitivity of heart rate was significantly attenuated in both models of renovascular hypertension. Beta-1-adrenergic blockade with MET did not alter this difference (Figure 3A-B). Following ATR, baroreflex sensitivity of heart rate was blunted in all groups. AT1-receptor blockade with Irb restored baroreflex sensitivity of heart rate in 2K1C animals (Figure 3C-D).

### Elevated sympathetic tone in both models of renovascular arterial hypertension (2K1C, 1K1C)

**Figure 4:**
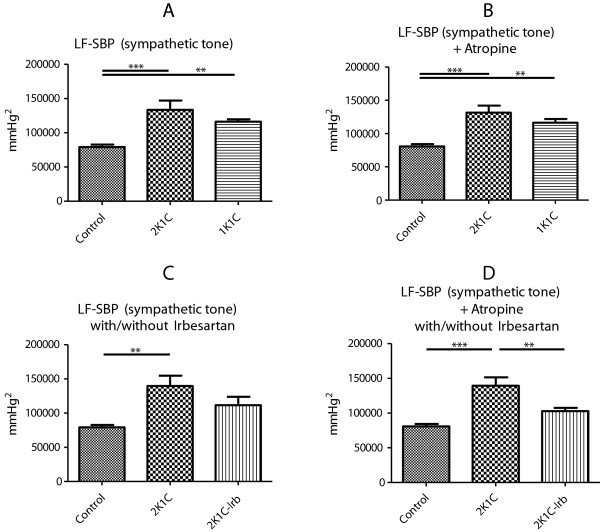
Sympathetic tone (LF-SBP) in controls (Control), 1K1C, and 2K1C animals (A); in Control, 1K1C, and 2K1C animals following ATR (B); in Control, and in 2K1C animals with and without irbesartan (Irb) treatment at baseline (C) or following ATR (D).

In comparison to controls, both models of renovascular hypertension (2K1C, 1K1C) presented with an elevated sympathetic nervous system tone as determined by power spectral analysis of systolic blood pressure. In 2K1C as well as in 1K1C animals, sympathoactivation persisted after ATR application (Figure 4A-B). In the 2K1C model, sympathetic tone was significantly higher without Irb and returned to normal levels with Irb treatment. This effect was maintained after ATR injection (Figure 4C-D).

### Parasympathetic tone is reduced in the model of renovascular hypertension with preserved kidney mass (2K1C)

**Figure 5:**
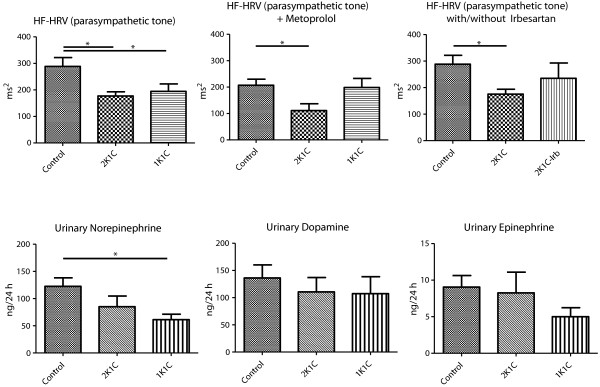
Upper panel: parasympathetic tone (HF-HRV) in controls (Control) at baseline and following MET; in addition, parasympathetic tone (HF-HRV) in Control, and in 2K1C animals with and without irbesartan (Irb) treatment; lower panel: 24-hour urinary excretion of norepinephrine, epinephrine, and dopamine in Control, 1K1C and 2K1C animals.

Parasympathetic tone, as determined by the cumulative intensity of HF-HRV, was significantly attenuated in both models of renovascular hypertension. Beta-adrenergic blockade with MET confirmed the significant attenuation of parasympathetic tone in 2K1C animals. An AT1-receptor blockade with Irb prevented the significant attenuation of parasympathetic tone in comparison to controls (Figure 5, upper panel).

As an alternative assessment of parasympathetic tone, LV-HRV was determined (Table 3). Following metoprolol, LF-HRV data confirmed the suppression of parasympathetic tone in 2K1C animals in comparison to controls (p < 0.05 in post-hoc test). However, when using LF-HRV as a surrogate for parasympathetic tone, there was no significant change in parasympathetic tone following AT1-receptor blockade in 2K1C animals (Table 4). In addition to the LF-HRV data, HF-SBP data is provided for all groups in Table 3 and Table 4.

**Table 3:** Supplemental power spectral data, i.e. cumulative intensity of high-frequency band of systolic blood pressure (HF-SBP) and of low-frequency band of heart rate (LF-HRV), is shown in normal control animals following sham surgery as well as in hypertensive animals (2-kidney-1-clip (2K1C); 1-kidney-1-clip (1K1C))

	**Normal controls**	**2K1C**	**1K1C**	**P**
LF-HRV	411.1 ± 170.8	282.8 ± 125.2	298.9 ± 133.2	0.15
LF-HRV following Metoprolol	328.0 ± 103.3	174.7 ± 105.2	271.1 ± 90.2	< 0.05
LF-HRV following Atropine	151.0 ± 111.1	90.4 ± 43.7	86.2 ± 68.5	< 0.05
HF-SBP	103208 ± 12697	154311 ± 35205	159123 ± 24991	<0.001
HF-SBP following Metoprolol	102988 ± 12776	148740 ± 28677	165959 ± 31325	<0.001
HF-SBP following Atropine	134689 ± 25981	178285 ± 35102	189734 ± 30970	<0.01

Statistical analysis was performed among groups.

**Table 4:** Supplemental power spectral data, i.e. cumulative intensity of high-frequency band of systolic blood pressure (HF-SBP) and of low-frequency band of heart rate (LF-HRV), is shown in normal control animals following sham surgery and in hypertensive animals (2-kidney-1-clip (2K1C)) with and without irbesartan (Irb) treatment

	**Normal controls**	**2K1C**	**2K1C-Irb**	**P**
LF-HRV	411.1 ± 170.8	303.9 ± 128.1	328.0 ± 170.5	0.36
LF-HRV following Metoprolol	328.0 ± 103.3	187.3 ± 120.6	439.1 ± 237.3	0.07
LF-HRV following Atropine	151.0 ± 111.1	96.7 ± 52.0	131.7 ± 83.7	0.36
HF-SBP	103208 ± 12697	157333 ± 36560	134874 ± 35018	<0.01
HF-SBP following Metoprolol	102988 ± 12776	154627 ± 28676	147225 ± 32901	<0.01
HF-SBP following Atropine	134689 ± 25981	182574 ± 42032	165463 ± 59555	0.11

Statistical analysis was performed among groups.

Collectively, renovascular hypertension with preserved kidney mass (2K1C) associates with a lower parasympathetic tone when compared to normal controls, which is reversed, at least partly, by AT1 receptor blocker treatment.

### Urinary catecholamines do not reflect sympathoexcitation in renovascular hypertension

Urinary norepinephrine excretion was significantly reduced in renovascular hypertension with reduced kidney mass (1K1C) when compared to normal controls (Figure 5, lower panel). In renovascular hypertension with preserved kidney mass, norepinephrine excretion was not different when compared to 1K1C animals or normal controls. This finding contrasts telemetric power spectral data of systolic blood pressure suggesting a state of sympathoactivation in both models of hypertension (1K1C, 2K1C), irrespective of residual kidney mass.

### Rho-kinase inhibition improves baroreflex sensitivity of heart rate in renovascular hypertension with reduced kidney mass

**Figure 6:**
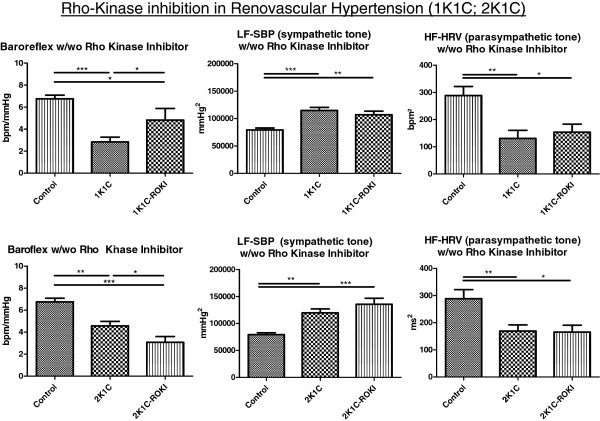
Upper panel: baroreflex sensitivity of heart rate in controls (Control), and in 1K1C animals with or without Rho-Kinase inhibitor (ROKI) treatment (SAR407899A uptake: 10.9 ± 3.5 mg/kg/d); lower panel: baroreflex sensitivity of heart rate in Control, and in 2K1C animals with or without ROKI treatment (SAR407899A uptake: 10.5 ± 2.4 mg/kg/d).

As demonstrated in Figure 6, baroreflex sensitivity of heart rate was improved by ROKI treatment in renovascular hypertension with reduced renal mass (1K1C). In contrast, ROKI treatment led to an attenuation of baroreflex sensitivity of heart rate in renovascular hypertension with preserved kidney mass (2K1C). These differential treatment effects of SAR407899A did, however, not translate into alterations of sympathetic or parasympathetic tone (Figure 6).

## Discussion

In this study, power spectral analysis of systolic blood pressure [26] and urinary catecholamines [33] were determined to gauge sympathetic tone. In addition, vagal tone was assessed using power spectral analysis of heart rate [26,30]. Slope data of concomitant, spontaneous pulse-interval and blood-pressure changes were gathered to estimate baroreflex function [31].

The results suggest that therapeutic interventions in renovascular hypertension may depend on residual renal mass. As shown for Rho-kinase inhibition, a beneficial effect on baroreflex function only emerged in the 1K1C model, but not in the 2K1C model of renovascular hypertension. This finding may potentially be due to the oxidative stress in the 1K1C model which leads to a more profound Rho A/ROCK activation [34-36]. In addition, Rho-Kinase inhibition may increase nitric oxide availability in hypothalamic centres of baroreflex regulation similar to the heart failure situation [20] which, in turn, improves baroreflex sensitivity of heart rate. Although baroreflex sensitivity of heart rate improved upon Rho-kinase inhibition in the 1K1C model of hypertension, this change did not translate into a reduction of the sympathetic or an increase in parasympathetic tone. This observation was unexpected given that carotid baroreflex function and/or baroreflex-dependent central nervous system regulations affect both sympathetic and parasympathetic tone [37,38].

In renovascular hypertension with preserved kidney mass (2K1C model), AT1-receptor blockade improved baroreflex sensitivity of heart rate. These data are supported by observations in humans with “essential” hypertension [39]. In addition, in response to AT1 receptor blockade, sympathetic tone normalized in the 2K1C model of hypertension which is in line with previous observations [40]. However, Rho-kinase inhibition was not shown to improve baroreflex sensitivity of heart rate in the 2K1C model. This difference to the 1K1C model may be due to lower nitric oxide availability in hypothalamic centres of baroreflex regulation in the 2K1C model of hypertension. Additional studies on the effect of Rho kinase inhibition are therefore needed to dissect the specific regulations of autonomic nervous system tone in different models of renovascular hypertension.

Concerning the parasympathetic tone, different results have been obtained for the renovascular hypertension models with and without reduced kidney mass (1K1C; 2K1C) as well. 2K1C animals showed a significantly depressed parasympathetic tone which persisted after beta-adrenergic blockade. As novel findings, AT1-receptor blockade with Irb significantly increased parasympathetic tone, decreased resting heart rate, and restored baroreflex sensitivity of heart rate in renovascular hypertension with preserved kidney mass (2K1C). Baroreflex and heart-rate data are in line with published evidence from experimental renoparenchymal hypertension [41]. Given the tremendous effect of baroreflex activating therapies in refractory hypertension [42], the beneficial role of AT1-receptor blockade with regard to baroreflex function deserves further attention in studies. In the present study, AT1 blockade was not applied to 1K1C animals because of the risk of kidney failure. Previous ultra-short term studies with losartan have not shown an improved baroreflex function in this volume-dependent model of renovascular hypertension (1K1C) [43].

As another main result, both models of renovascular arterial hypertension exhibited a state of sympathoactivation as detected by power spectral analysis of blood pressure. Thus, previous results obtained with selective blockade (atropine and propranolol) experiments on heart rate were confirmed [8,9]. For renovascular hypertension with preserved kidney function (2K1C), a prevalent sympathoactivation was also reported in a recent study using the same methodology in rats as used in the present study [44]. Interestingly, in that study, baroreflex depression occurred almost instantaneously upon induction of Goldblatt hypertension (2K1C). In the present study, the level of sympathoactivation was similar between 1K1C and 2K1C animals, despite the different hormonal cause of hypertension [12] and the different volume state [45]. Apart from renovascular hypertension, a state of sympathoactivation was found in patients with “essential” arterial hypertension [46-49] and in chronic heart failure. In the latter, effects on the central nervous system by angiotensin II were postulated [50,51]. For the 1K1C model, the detailed mechanism of sympathoactivation, however, is still unclear. In 1K1C animals, renin release is known to be similar to normal controls [12]. Therefore, systemic RAAS activation cannot be a cause of sympathoactivation under this condition. However, the brain “ouabain” and/or the brain renin-angiotensin system may become pertinent for sympathoactivation in renovascular hypertension with reduced kidney mass possibly through a sodium-dependent mechanism [52]. In addition, kidney mass reduction and renal artery stenosis may reduce urinary norepinephrine excretion as shown here (Figure 6). Diminished excretion and/or attenuated catecholamine degradation in the kidney [53-55] may lead to catecholamine accumulation and, potentially, sympathoactivation. As a limitation of the present study, plasma catecholamines were not determined. Uremic toxins are unlikely to play a sympathoactivating role in the 1K1C model because glomerular filtration was shown to be reduced by only 30% [56].

## Conclusions

As main results, sympathetic tone (with or without vagal blockade) was found to be increased, whereas baroreflex sensitivity of heart rate was depressed in models of renovascular hypertension, irrespective of residual renal mass. Differential results relate to parasympathetic tone (with or without beta 1-adrenergic blockade) that was depressed in the 2K1C model only. In addition, left ventricular hypertrophy was present in experimental renovascular hypertension with reduced renal mass (1K1C) only. Renal norepinephrine excretion was reduced in the 1K1C model exclusively. Hypothetically, renal reduced catecholamine excretion and/or impaired renal catecholamine degradation may be considered as mechanisms of sympathoactivation in the 1K1C model. Finally, Rho-kinase inhibition improved baroreflex function solely in experimental renovascular hypertension with reduced renal mass (1K1C), whereas AT1 blockade improved baroreflex sensitivity of heart rate in renovascular hypertension with preserved kidney mass (2K1C). Taken together, Rho-kinase inhibition might be an additive strategy to improve survival in renovascular hypertension with reduced renal mass, whereas low-dose AT1 blockade might be a therapeutic choice in all other cases of renovascular hypertension.

## Competing interests

The authors declare that they have no competing interests.

## Authors’ contributions

RUP made substantial contributions in study conception and design and the acquisition of data. He performed experiments and drafted the manuscript. SB and OJ collected data, performed experiments and provided substantial input in data interpretation and analysis. RPB conceived the study, contributed to study design and was involved in drafting the manuscript. All authors gave final approval to the version to be published.
